# Making immotile sperm motile using high-frequency ultrasound

**DOI:** 10.1126/sciadv.adk2864

**Published:** 2024-02-14

**Authors:** Ali Vafaie, Mohammad Reza Raveshi, Citsabehsan Devendran, Reza Nosrati, Adrian Neild

**Affiliations:** Department of Mechanical and Aerospace Engineering, Monash University, Clayton, Victoria 3800, Australia.

## Abstract

Sperm motility is a natural selection with a crucial role in both natural and assisted reproduction. Common methods for increasing sperm motility are by using chemicals that cause embryotoxicity, and the multistep washing requirements of these methods lead to sperm DNA damage. We propose a rapid and noninvasive mechanotherapy approach for increasing the motility of human sperm cells by using ultrasound operating at 800 mW and 40 MHz. Single-cell analysis of sperm cells, facilitated by droplet microfluidics, shows that exposure to ultrasound leads to up to 266% boost to motility parameters of relatively immotile sperm, and as a result, 72% of these immotile sperm are graded as progressive after exposure, with a swimming velocity greater than 5 micrometer per second. These promising results offer a rapid and noninvasive clinical method for improving the motility of sperm cells in the most challenging assisted reproduction cases to replace intracytoplasmic sperm injection (ICSI) with less invasive treatments and to improve assisted reproduction outcomes.

## Teaser

A noninvasive ultrasound-based mechanotherapy method tailored for applications in assisted reproduction enhances sperm motility.

## INTRODUCTION

Sperm motility is central to natural fertilization (*1*), considerably influencing both the likelihood of successful fertilization (*2*) and offspring health (*3*). Every 10% increase in sperm motility leads to an increase of 8% in pregnancy rate (*4*). With respect to assisted reproduction, high sperm motility allows for treatment options that are less invasive (*5*), have higher fertilization, implantation, and pregnancy rates (*6*, *7*); pose lower risks to offspring health (*3*); and are cost-effective (*8*–*10*). For example, in intracytoplasmic sperm injection (ICSI), sperm motility is correlated with embryo quality (*11*), and the success rate of fertilization cycles increases from 20 to 48% with the injection of motile sperm rather than immotile cells, improving the pregnancy rate by ~25% (*12*). Low sperm motility also precludes the use of less invasive treatment options, which seek to mimic the natural motility-based sperm selection process. Chemical drugs such as pentoxifylline, theophylline, and other phosphodiesterase inhibitors have been used to enhance sperm motility (*13*, *14*). These drugs increase the intercellular level of cyclic adenosine monophosphate to induce flagellum protein phosphorylation and stimulate motility (*13*, *15*). However, the embryotoxicity of drugs such as pentoxifylline, their potentially harmful effects on sperm longevity, and the need for multistep washing that introduces reactive oxygen species and DNA damage (*16*–*18*) have been major concerns that considerably limited their applications in fertility clinics (*13*, *19*).

Sperm acquires motility after undergoing the process known as spermiogenesis in the epididymis (*20*). The sperm cell uses two main metabolic pathways to generate adenosine triphosphate (ATP) energy units for motility. The first pathway is oxidative phosphorylation (OXPHOS), which comprises a series of oxidation-reduction reactions occurring in sperm mitochondria accompanied by electron transfer through the electron transport chain that results in ATP synthesis (*21*). The second is glycolysis in the principal piece (a section of the tail) (*22*), which refers to glucose breakdown into three-carbon compounds called pyruvate that results in energy production in the form of ATP (*23*). Depending on the transition stage and species (*24*, *25*), sperm activate either of these two pathways during its journey toward the egg to regulate its motility and metabolic activity levels. Mitochondrial OXPHOS as the main pathway for sperm functionality and motility (*26*) is regulated through mechanisms in which mitochondrial calcium (Ca^2+^) transmission has a key role to maintain the required energy level for motility (*27*, *28*). Abnormal sperm mitochondria condition including low count of mitochondrial gyres (*29*) and its disorganization (*30*) leads to sperm dysfunction and, in some cases, results in severe asthenozoospermia (poor sperm motility) (*26*, *31*). Accordingly, the enhancement of mitochondrial metabolism and subsequent improvement in sperm motility could offer new treatment opportunities.

Acoustofluidic systems that use surface acoustic waves (SAWs) provide powerful biocompatible tools for cell manipulation (*32*) with applications in single-cell analysis (*33*) and tissue engineering (*34*). In SAW systems, the acoustic waves propagate from a piezoelectric substrate into a fluid volume, generating nonlinear acoustic radiation forces that cause the displacement of the particles or cells allowing patterning and sorting (*35*). A second nonllinear effect is acoustic streaming, which causes a steady bulk flow of fluid (*36*), causing microfluidic mixing (*37*). Aside from these physical effects, SAWs have also been used to alter the behavior of adherent cells to inhibit surface attachment and decrease cell spreading while increasing the rate of metabolic activity (*38*). This acoustic-based mechanotherapy approach has been shown to increase human and bull sperm motility at a population level (*39*). However, the acoustic radiation force and streaming-induced effects displace the sperm considerably (*40*) and render the tracking of individual sperm before and after exposure highly problematic. Droplet microfluidics facilitates high-throughput evaluation of individual cells in an isolated microenvironment (*41*). In droplet microfluidics, immiscible fluids are merged to create a compartmentalized two-phase flow. Channel geometries and flow speeds can be used to vary the size and rate of production of the droplets (*42*). This size control has been used to create curved soft interfaces to indicate how the increasing curvature of the soft epithelial tissue in vivo promotes sperm capacitation and fertilization competence (*43*). However, it is the compartmentalized nature of each droplet that has been exploited most widely to enable single-cell level biology (*44*), most notably in single-cell RNA sequencing, whereby individual cells are lysed in each droplet (*45*). By isolating sperm within droplets, although acoustic streaming causes some movement of the droplets and there is flow within the droplet, individual cells can easily be identified before and after exposure.

In this work, we present a droplet acoustofluidic platform for retrieving and improving human sperm motility at the single-cell level. Our single-cell analysis results show that sperm with low initial motility are those which see the largest change in motility after ultrasonic exposure (at 800 mW and 40 MHz). The percentage of nonprogressive sperm in a sample reduces from 36% to just 10% after 20 s of ultrasonic treatment, promising new treatment opportunities for asthenozoospermia samples with poor motility. This increase in motility is even more profound when treating immotile sperm, rendering 34% of live immotile sperm motile after exposure. Of these, 24% of the treated cells exhibit progressive motility and a significant increase (*P* < 0.0001) in swimming velocity after exposure, while the other 10% show twitching behavior. This latter outcome offers a tremendous potential for improving clinical outcomes by treating complete asthenozoospermia or testicular samples. Identifying live cells (without the need for staining or chemical treatment) in testicular sperm samples by inducing motility is beneficial for ICSI. This noninvasive method for enhancing sperm motility can be used in the most challenging ART cases that currently suffer from low success rates (*46*).

## RESULTS

**Fig. 1.**
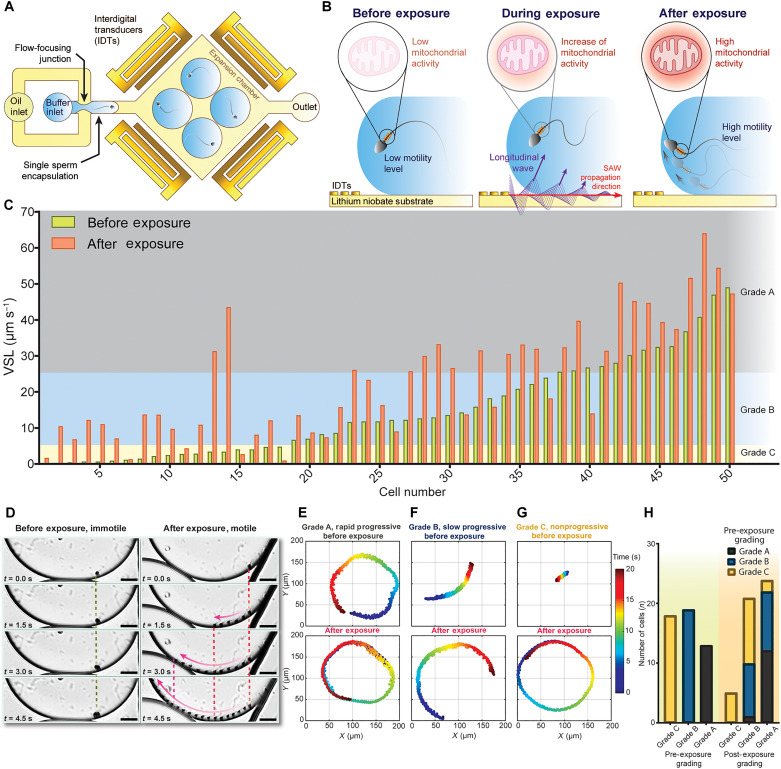
Rendering immotile sperm motile at the single-cell level using ultrasound exposure at 40 MHz and 800 mW. (**A**) Schematic of the developed droplet acoustofluidic platform for analyzing the effect of ultrasound on an individual sperm cell. (**B**) SAWs generated by the interdigital transducers (IDTs) couple from the LN surface into the droplet to enhance sperm motility by increasing the intercellular level of metabolic activity. (**C**) VSL values of 50 sperm cells before and after exposure to ultrasound indicating a jump from grade C (nonprogressive, VSL < 5 μm s^−1^) motility level before exposure to grade B (slow progressive, 5 ≤ VSL < 25 μm s^−1^) and grade A (rapid progressive, 25 μm s^−1^ ≤ VSL) motility levels after exposure. The background highlight shows the VSL range, which belongs to each motility grade. (**D**) Representative overlaid image sequence of an immotile sperm rendered motile upon exposure to ultrasound. Scale bar, 20 μm. Representative swimming trajectories of sperm cells with pre-exposure motility of (**E**) grade A, (**F**) grade B, and (**G**) grade C before (top) and after (bottom) exposure to ultrasound. The color corresponds to time in seconds. (**H**) Comparison of the number of sperm in each motility grade before and after exposure to ultrasound. Color indicates the initial motility grade before exposure. In (C) and (H), a total of 50 cells from three independent experiments with three biologically independent human samples were used.

To enable tracking and analyzing sperm motility over time at the single-cell level, individual sperm was encapsulated in microdroplets (Fig. 1A) and imaged for 20 s before and after exposure to ultrasound at 40 MHz and 800 mW. During exposure, SAWs couple from the lithium niobite (LN) substrate into the fluid; hence, ultrasound impinges on the sperm isolated in the droplets as schematically shown in Fig. 1B. The sperm cells were categorized into three motility grades based on their straight-line swimming velocity (VSL; see Materials and Methods) in accordance with the World Health Organization (WHO) (*47*) guidelines. These motility grades are defined as rapid progressive (grade A) with VSL ≥ 25 μm s^−1^, slow progressive (grade B) with 5 ≤ VSL < 25 μm s^−1^, and nonprogressive or immotile (grade C) with VSL < 5 μm s^−1^. Figure 1C shows the pre- and post-exposure VSL values of individual sperm cells included in this analysis. For more than 86% of live sperm cells (*n* = 50; fig. S1), the curvilinear swimming velocity (VCL; sperm instantaneous swimming velocity along its trajectory) was improved upon exposure to ultrasound (see movie S1). Figure 1D shows a sequence of images for an immotile sperm (grade C) with just a twitching motility pre-exposure that was rendered fully motile (grade A, VSL = 31.4 μm s^−1^) after exposure to ultrasound. The VCL of this initially immotile sperm was increased to 31.1 μm s^−1^ after exposure to swim a 35-fold longer distance in 4.5 s (an increase from 4 to 140 μm), with an accompanying 399 and 310% increases in average path velocity (VAP) and linearity (LIN), respectively.

Exposure to ultrasound not only increased the swimming velocity of sperm but also 59% of grade C and B cells moved to a higher motility grade after exposure to ultrasound (Fig. 1, C to H), with 2 of 18 grade C cells becoming grade A after exposure. Figure 1H indicates the distribution of cells in each grade before and after exposure (color coded based on their pre-exposure motility grades). Initially, 36% of sperm were nonprogressive, 38% were slow progressive, and 26% were rapid progressive. After exposure, the cells were redistributed into a population with only 10% nonprogressive, 42% slow progressive, and 48% rapid progressive sperm. Notably, 61 and 11% of grade C sperm moved to grade B and A, respectively, and 47% of grade B cells moved to grade A. We also evaluated the DNA integrity and viability of sperm cells after exposure to ultrasound as compared with the initial sample (fig. S2 and table S1). The results indicated no significant changes in DNA integrity or viability after exposure, indicating the biocompatibility and noninvasiveness of our method.

**Fig. 2.**
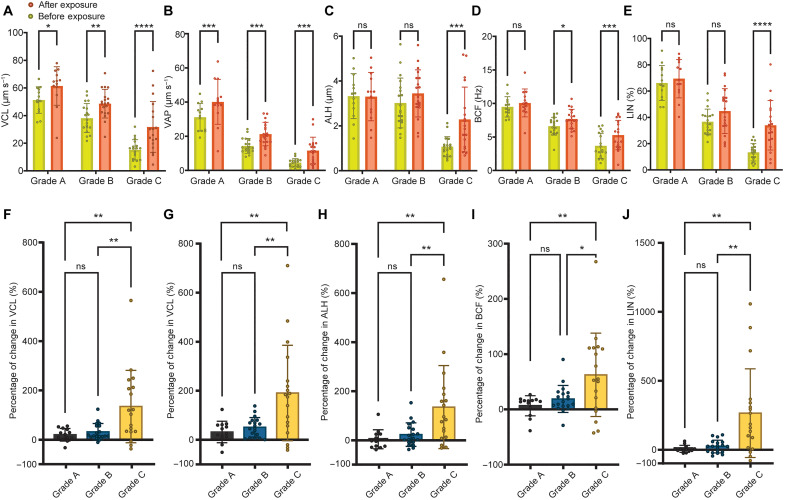
Comparison of the change in motility parameters after exposure to ultrasound as a function of initial pre-exposure motility grades. Within-group comparisons of (**A**) VCL, (**B**) VAP, (**C**) ALH displacement, (**D**) BCF, and (**E**) LIN before and after exposure. In-between group comparisons of the percentages of change in (**F**) VCL, (**G**) VAP, (**H**) ALH, (**I**) BCF, and (**J**) LIN. Values are reported as means ± SD (tables S4 and S5). For each motility grade, *n* ≥ 13 sperm from three independent experiments with three biologically independent human samples were used. Statistical significance for within-group comparisons was determined using two-way analysis of variance (ANOVA)–matched values with Bonferroni’s multiple-comparison test, and for in-between group comparisons, it was determined using ordinary one-way ANOVA with Tukey’s multiple-comparison test (*P ≤ 0.05, **P ≤ 0.01, ***P ≤ 0.001, and ****P ≤ 0.0001; ns denotes not significant).

To quantify which motility grade benefits the most from the boost caused by ultrasound, we evaluated the change in motility parameters for cells grouped based on their pre-exposure VSL values (Fig. 2). Within-group comparisons (Fig. 2, A to E) reveal that grade C sperm is the only grade that experienced a statistically significant boost to all of the motility parameters (table S2). Grade C sperm experienced an increase of 109% in VCL (from 15 to 32 μm s^−1^), 159% in VAP (from 5 to 12 μm s^−1^), 112% in amplitude of lateral head (ALH) (from 1.1 to 2.3 μm), 44% in beat cross frequency (BCF) (from 3.7 to 5.3 Hz), and 153% in LIN (from 13 to 34%) after exposure as compared with pre-exposure values (table S3). The results suggest that grade C sperm improve their progressive motility after exposure, potentially by increasing their flagellar wave amplitude and beating frequency while reducing the yaw in their swimming trajectory to swim straighter. Grade B sperm showed a statistically significant boost to VCL (by 27%, from 38 to 49 μm s^−1^), VAP (by 48%, from 14 to 21 μm s^−1^), and BCF (by 16%, from 6.6 to 7.7 Hz) after exposure; however, the increase in ALH and LIN was not statistically significant. Grade A sperm cells showed a statistically significant increase only in VCL (an increase of 20% from 51 to 61 μm s^−1^) and VAP (an increase of 29% from 31 to 40 μm s^−1^). This change in motility parameters suggests that the increase in swimming velocity of grade B sperm is potentially attributed to a faster movement along the average path (higher beating frequency after exposure), while for grade A sperm, the beating amplitude and frequency may not change, but the flagellar wavelength may increase to facilitate an increase in sperm progressive velocity (*48*). Between group comparison of the boost caused by ultrasound (Fig. 2, F to J) clearly indicates that poorly motile or even immotile grade C sperm benefit the most from ultrasound exposure. Specifically, grade C cells, on average, exhibited up to 11- and 38-fold higher improvement in motility parameters as compared with grade B and grade A sperm cells, respectively. Collectively, a significant boost was observed, on average, when comparing pre- and post-exposure motility parameters (fig. S3). In addition, to account for the spontaneous sperm motility changes, we analyzed the variations in the motility of individual sperm over 20 min before exposure, with intervals of 5 min. At the end of this pre-exposure period, sperm cells were exposed to ultrasound, and the post-exposure motility was subsequently analyzed (fig. S4). The results indicate that, despite the spontaneous motility variations over the 20-min pre-exposure period, the average pre-exposure sperm motility remained stable. However, after exposure to ultrasound, a significant boost in VCL was observed.

**Fig. 3.**
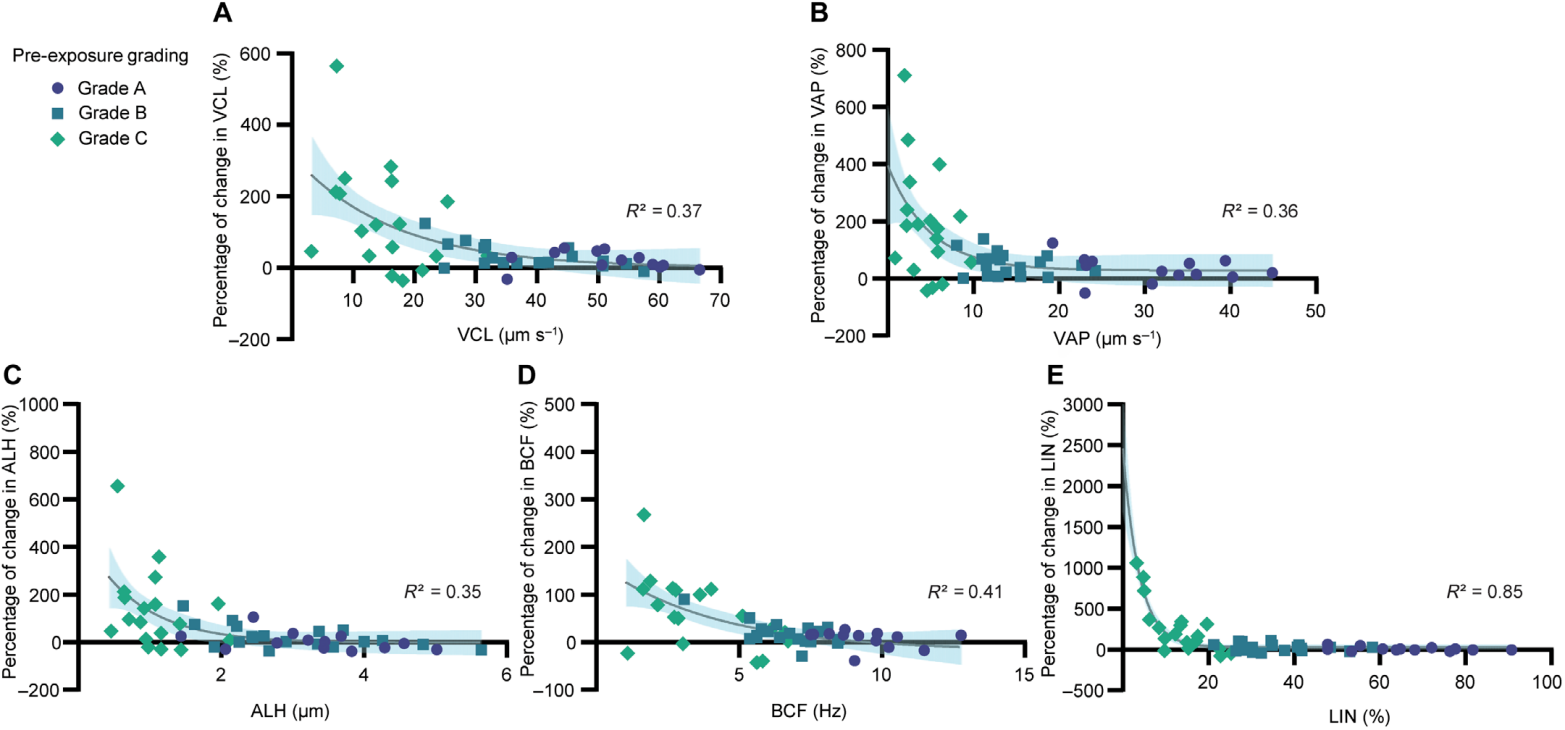
Single-cell level boost caused by ultrasound. Percentage of change in (**A**) VCL, (**B**) VAP, (**C**) ALH, (**D**) BCF, and (**E**) LIN of each cell after exposure versus the initial values before exposure. Each data point is an individual sperm (*n* = 50, from three independent experiments using three biologically independent human samples), and the symbol indicates their initial pre-exposure motility grade. The continuous line indicates the corresponding one phase decay least square fit (nonlinear regression), and the highlighted area for each graph represents the 95% confidence band.

Figure 3 shows the boost caused by ultrasound at the single-cell level. The results indicate a saturating behavior for the high motility ranges (pre-exposure VCL ≥ 50 μm s^−1^) with a limited percentage of increase (<65.5%; table S6), which suggests that there is a nonlinear trend with respect to the initial motility of sperm governing the effect of ultrasound. Despite exhibiting the strongest boost, the data points are more scattered for grade C cells than grade B and grade A cells, potentially due to the existence of highly immotile cells with severe motility defects and structural abnormality among grade C cells (*49*). Comparing our nonlinear (Fig. 3) to the linear regression analysis results (fig. S5), nonlinear trend curves provide higher *R*^2^ (coefficient of determination) values and hence better fit to the data points. Linear fits indicate statistically significant negative slope regression lines (fig. S5), confirming that poorly motile or immotile sperm experience a higher increase in the motility parameters compared to progressively motile sperm. Although the *R*^2^ values suggest moderate precisions for the models, it is important to note that models with relatively low *R*^2^ values can still have clinical utility and relevance (*50*).

**Fig. 4.**
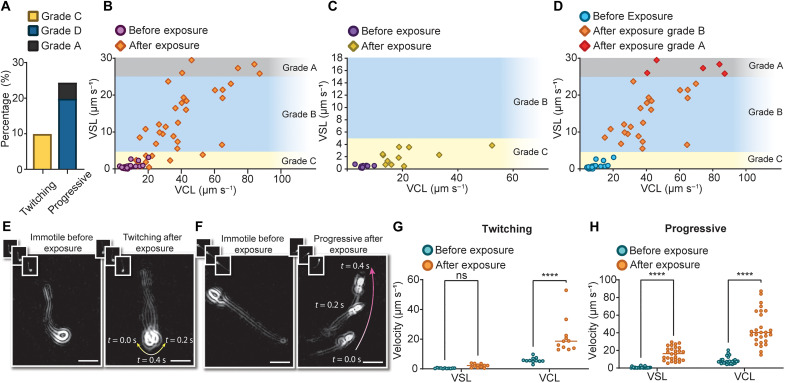
Effect of ultrasound on live immotile grade C sperm. (**A**) Percentage of sperm rendered twitching or progressive with respect to the total number of live immotile sperm (*n* =111, from six independent experiments using three biologically independent human samples). The color corresponds to post-exposure motility grades as shown in the legend. Post-exposure VSL versus VCL distributions for immotile cells (**B**) rendered motile (*n* = 38), (**C**) ended up as twitching (*n* = 11), and (**D**) ended up as progressive (*n* = 27) after exposure. Overlay images of representative initially immotile sperm rendered (**E**) twitching and (**F**) progressive after exposure composed of three frames of 200-ms intervals as shown in the inset. Scale bars, 10 μm. Comparison between before and after exposure values of VSL and VCL of (**G**) twitching and (**H**) progressive cells (table S2). Statistical significance was determined using two-way ANOVA-matched values with Bonferroni’s multiple-comparison test (****P ≤ 0.0001).

To demonstrate the capability of the ultrasound approach for detecting live sperm in a challenging sample, we evaluated how live but completely immotile sperm react to ultrasound. To that aim, a sample of immotile sperm was prepared and stained, with live sperm exhibiting green fluorescence, using the sperm viability kit (see Materials and Methods). These stained immotile sperm were isolated in droplets and exposed to ultrasound (at a power of 800 mW and frequency of 40 MHz). After exposure, 34% of the live immotile sperm exhibited some form of motility (Fig. 4), with no significant difference in responses between samples from different donors (fig. S6). For the remaining 66% of live immotile sperm, while no noticeable flagellar deformation was observed for the majority of cells (similar to dead cells), 25% of them showed flagellar deformation during exposure, but they did not become motile or show twitching after exposure (see movie S2). This indicates the unique capability of our noninvasive approach for dealing with challenging samples with complete asthenozoospermia or testicular samples from azoospermia patients (with no sperm in the ejaculate). As shown in Fig. 4 (A to D), among the 111 analyzed live immotile cells, more than 25% were rendered as progressive after exposure with a significant increase in both VCL (*P* < 0.0001) and VSL (*P* < 0.0001), and 9% remained nonprogressive but with induced twitching motility. The exposed sperm cells showed a VSL between 0.5 and 29.5 μm s^−1^ and a VCL in the range of 12.9 to 87.2 μm s^−1^ after exposure (Fig. 4, B to D). The sperm rendered twitching (Fig. 4E) showed oscillatory movements rather than progressive motion, while the sperm rendered progressive (Fig. 4F) started moving forward after exposure. In both cases, these sperm were identified as live before exposure through the use of a fluorescent dye; however, after exposure, the movement of the cells means that visual assessment without the need for dye is sufficient to identify these cells as live—a promising noninvasive tool to potentially select viable sperm from testicular samples for use in ICSI.

The 25% of live immotile sperm rendered progressive were split into 5% grade A and 20% grade B cells after exposure (Fig. 4, A and D), with their post-exposure VSL ranging from 5.5 to 29.5 μm s^−1^ and their VCL ranging from 14.9 to 87.2 μm s^−1^ (Fig. 4, D and H). Sperm cells with induced twitching motility had VSL in the range of 0.5 and 3.8 μm s^−1^ and VCL between 12.9 and 52.9 μm s^−1^ (Fig. 4, C and G). Despite a statistically nonsignificant boost to VSL (*P* = 0.94; Fig. 4G), the twitching category experienced a statistically significant boost to VCL (*P* < 0.0001) by 268%. On the other hand, sperm rendered progressive showed a statistically significant boost to both VSL and VCL by 2348 and 435%, respectively (Fig. 4H).

**Fig. 5.**
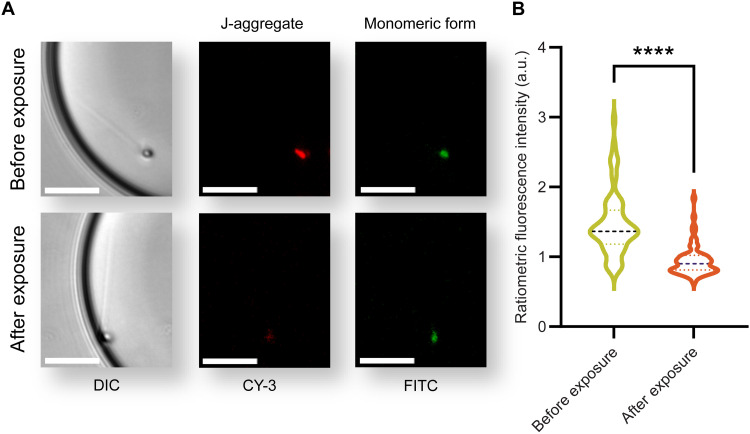
Sperm MMP change upon ultrasound exposure. (**A**) Representative images of an example individual sperm labeled by JC-1 dye to evaluate the fluorescence intensity of red J-aggregates and green monomers before and after exposure using CY3 and fluorescein isothiocyanate (FITC) filters, respectively. The images were contrast-adjusted for clarity. Scale bars, 20 μm. (**B**) Comparison between the ratiometric fluorescence intensity of red J-aggregate to green monomers before and after exposure to ultrasound (*n* = 58, from three independent experiments using three biologically independent human samples). Values are reported as means ± SD (table S7). Statistical significance was determined using paired *t* test (****P ≤ 0.0001). DIC, differential interference contrast; a.u., arbitrary units.

To resolve the underlying biomechanism behind the motility boost caused by ultrasound, we evaluated the regulation of mitochondrial function by monitoring the mitochondrial membrane potential (MMP) levels (see Materials and Methods). MMP is the electrical gradient across the inner mitochondrial membrane (*51*) that energizes the synthesis of ATP by driving protons back into the matrix through mitochondrial ATP synthase (*52*). In addition to protons, MMP drives the transport of other charged compounds across the inner membrane, which is necessary for mitochondrial function (*53*). A change in MMP level indicates an alteration in the energy supply within the cell that can affect ATP production and cell motility. Figure 5 shows the ratiometric analysis of red (high MMP) to green (low MMP) fluorescence intensities. The results reveal that after exposure to ultrasound at 40 MHz and 800 mW, the increase in sperm motility is accompanied by a statistically significant reduction in the MMP level, indicating that exposure to ultrasound regulates mitochondrial function.

## DISCUSSION

We used droplet microfluidics to study the effect of ultrasound on retrieving human sperm motility at the single-cell level. Our results reveal that exposure to ultrasound leads to an increase in sperm motility parameters with low motility sperm benefit the most from this effect (up to 266% improvement in motility). Exposure to the ultrasound causes slow and rapid progressive sperm (grades B and A, respectively) to swim more than 32% faster along the average path and become more progressive, potentially by increasing their beating frequency and/or flagellar wavelength while maintaining the same beating amplitude. For nonprogressive sperm (grade C), a significant increase in all motility parameters occurs mainly due to a higher flagellar wavelength, amplitude, and beating frequency, after exposure. Note that ultrasound exposure increased the temperature in the sperm chamber by approximately 1.5°C. However, the boosting effect was comparable to that in cells exposed to ultrasound over a Peltier cooling platform, where the temperature remained relatively stable (*39*). Moreover, the motility boost due to ultrasound exposure occurs at the onset of exposure (see movie S3), while the temperature change happens 20 s after the exposure initiation.

When it comes to comparing the boost gained by different grades, results reveal that nonprogressive sperm experience a significantly higher boost to all motility parameters compared to progressive sperm, with saturation in the ultrasonic boost for initially high motility sperm. This saturation in the boost for high motility sperm can be attributed to a maximum limit for the mitochondrial ATP synthesis rate, which confines further motility boost by ultrasound (*54*). Post-exposure grading of sperm indicates that the majority of sperm were classified as grade A after exposure, increasing from 26% before exposure to 48% after exposure, while 72% of the grade C sperm moved to higher grades and became progressive after exposure. We also examined the effect of ultrasound on immotile sperm and observed that more than 34% of live immotile sperm are rendered motile after exposure, which can be subdivided into 25% progressive and 9% twitching cells. Our ultrasound platform provides a noninvasive approach to boost sperm motility, with no significant changes in sperm DNA integrity or viability after exposure (fig. S2). It is well known that sperm undergo fluctuations in their motile activities. The activation using ultrasound prompts an increase in motile activity, and these allows the identification of those cells capable of transitioning from an immotile to a motile state.

We also show that there is a reduction in the MMP level of sperm cells upon exposure to ultrasound (Fig. 5). Mitochondria use regulatory processes to balance between ATP demand and ATP production in accordance with cell function and environmental stresses (*55*, *56*). Several potential mechanisms can contribute to the reduction in MMP upon exposure to ultrasound. Ultrasound could enhance mitochondrial Ca^2+^ uptake through the activation of mechanosensitive ion channels (*57*) to adapt OXPHOS substrate supply for ATP needs (*27*, *58*) and contribute to maintaining motility (*59*–*62*). Depolarization of the MMP could also be explained by the reverse operation of the mitochondrial shuttle systems as a result of the increased rate of glycolysis in the principal piece upon exposure, which subsequently leads to the depolarization of the reduced form of nicotinamide adenine dinucleotide (NAD^+^):NAD^+^ redox potential (*63*, *64*) Another plausible explanation for the drop in MMP is the increase in the rate of ATP hydrolysis in the flagellum in response to ultrasound, which leads to a less negative ATP hydrolysis free energy and dissipation of the proton motive force (*65*, *66*).

Our motility boost results are promising for the application of this mechanotherapy method in assisted reproduction. The motility of the sperm in a patient’s sample determines the selection of the most appropriate therapy and considerably influences the success rate of the selected treatment cycle (*67*). Hence, being able to alter motility can potentially alter the selection of therapy type and the resulting outcomes toward the application of less invasive, more affordable options.

In in vitro fertilization (IVF), in which sperm and oocyte are co-incubated to establish fertilization, the concentration of motile sperm and type of sperm motility play decisive roles in fertilization outcomes. If motile sperm constitute less than 40% of total sperm, then a significantly higher rate of fertilization failure in IVF occurs (*68*), and when the concentration of progressively motile sperm exceeds 40%, it leads to a significant 15% increase in the fertilization rate (*69*). As the utilization of ultrasound not only increases the number of motile sperm by improving the motility of nonprogressive sperm but also drives motile sperm to swim more progressively, without detrimental effects on sperm DNA integrity and viability (*39*, *40*), this noninvasive technology can be integrated with IVF equipment to enhance the fertilization rate and increase successful outcomes. The increase in motility of nonprogressive sperm and rendering immotile sperm motile can also assist in crossing the threshold of at least 30% motile sperm, 15% of which have progressive motility (*70*) required for IVF, and hence allow more patients to be offered this less invasive approach to assisted reproduction.

With respect to treating immotile sperm by ultrasound, our results offer opportunities for the use of this mechanotherapy method for the treatment of clinical sperm samples from patients suffering from severe asthenozoospermia or testicular samples from azoospermia patients. In cases in which ICSI is required, twitching motility is a crucial indicator of sperm viability and the capability of establishing fertility (*12*). In this regard, selecting live sperm from testicular samples of azoospermia patients for use in ICSI is very challenging, and a significant failure of ICSI cycles was observed when only immotile sperm were recovered and injected (*71*). Accordingly, a variety of methods were developed to induce twitching motility to live immotile sperm for use in ICSI (*72*). However, these methods suffer from the lack of accuracy and reliability, invasiveness, use of chemicals, DNA damage, and high cost and time consumption (*72*). We showed that utilization of ultrasound at 40 MHz and 800 mW provides the capability to induce motility to more than one-third of live immotile sperm, which potentially enables a fast and noninvasive method for the selection of viable sperm from testicular samples.

Together, our findings show that ultrasound improves the motility of sperm cells by up to 266%. We reveal that by utilization of ultrasound operating at 800 mW and 40 MHz, 72% of nonprogressive sperm become progressive and more than 34% of live immotile sperm are rendered motile. Our method provides possible opportunities for using this technology in assisted reproduction for infertility cases caused by severe male factors such as asthenozoospermia. Ultrasound improves the motility of nonprogressive sperm more effectively, which can provide higher fertilization rates and successful outcomes in IVF. This method has the capability to noninvasively identify viable sperm by application of ultrasound only for a few seconds with potential use in ICSI to improve the most challenging assisted reproduction cases that currently suffer from low success rates. Future relevant studies include further exploration of motility boost at the mechanistic level, assessment of the integration capability of our mechanotherapy method with assisted reproduction platforms, and the evaluation of the effect of ultrasound on embryo.

## MATERIALS AND METHODS

### Sperm preparation

Fresh human semen samples were collected from healthy donors after 2 days of sexual abstinence with prior informed consent, and the study was approved by the Monash University Human Research Ethics Committee. A total of 16 biological replicates from three human donors, aged between 20 and 40 years old, with sperm concentrations ranging from 20 to 60 million sperm/ml, were used in this study. The fertility condition of the donors was not explicitly determined; however, donors had relatively stable semen parameters, and no exclusion criteria were applied. No significant difference between donors was observed (fig. S6). Fresh samples were incubated at 37°C for 30 min to liquefy. The swim-up method (*73*) was then used to isolate motile sperm from immotile cells and debris. Hepes-buffered salt solution (117 mM NaCl, 5.3 mM KCl, 2.3 mM CaCl_2_, 0.8 mM MgSO_4_, 1 mM NaH_2_PO_4_, 5.5 mM d-glucose, 0.03 mM phenol red, 4 mM NaHCO_3_, 21 mM Hepes, 0.33 mM Na-pyruvate, and 21.4 mM Na-lactate) supplemented with polyvinyl alcohol (1 mg ml^−1^) was used to run the swim-up assay. To achieve the optimal concentration for single-cell encapsulation in the microdroplets (*74*), the final sperm concentration in the sample was set to 1.4 million cells/ml. To collect poorly motile sperm as well, cells from the middle of the swim-up tube were collected. The deliberate inclusion of poorly motile and immotile sperm in the studied cells may have impacted the viability of the control sperm (refer to fig. S2).

### LIVE/DEAD assay

The LIVE/DEAD Sperm Viability Kit (Invitrogen, USA) was used to evaluate sperm viability (*75*) by staining live sperm with green fluorescence using SYBR-14 and dead sperm with red fluorescence using propidium iodide. To stain the cells, 5 μl of 20 μM SYBR-14 solution and 5 μl of the 2.4 mM propidium iodide solution were added to 1 ml of the prepared sperm sample and incubated at 37°C for 10 min in the dark. After the incubation, live and dead sperm were distinguished using fluorescent microscopy (Olympus IX83, Japan). Live sperm cells were identified by their green fluorescence (~516 nm) and imaged using a fluorescein isothiocyanate filter, while dead sperm emitted red fluorescence (~617 nm) and imaged using a CY3 filter.

### MMP assay

To analyze MMP, JC-1 (Invitrogen, USA), a carbocyanine cationic dye, was used to selectively stain the sperm mitochondria that shows potential-dependent accumulation across the electronegative interior of the inner mitochondrial membrane regardless of mitochondrial size, shape, and density (*76*, *77*). To stain the cells, 1.3 μl of 1.53 mM JC-1 solution was added to 1 ml of the sperm sample (final JC-1 concentration of 2 μM) at the concentration of 10 million cells/ml and incubated at 37°C for 20 min in the dark. Stained cells were imaged in red and green fluorescence using an inverted fluorescent microscope (Olympus IX83, Japan) at ×40 magnifications to evaluate MMP. In mitochondria with low MMP, JC-1 is in the monomeric form and emits green fluorescence (~525 nm), while a high MMP level leads to a reversible dye agglomeration and formation of J-aggregate that emits red fluorescence (~590 nm) (*78*). Accordingly, the recorded images were analyzed in ImageJ and the ratio of red to green fluorescence per cell was used to evaluate the MMP level (*76*, *77*). Carbonyl cyanide chlorophenylhydrazone at the concentration of 5 mM was used as the MMP-negative control to validate the JC-1 staining by disrupting the membrane potential of sperm cells stained by JC-1. When recording videos, before exposure, the fluorescence intensity of sperm mitochondria was also measured and the rate at which photobleaching was occurring was calculated and compensated for in the MMP intensity analyses.

### Device fabrication

The droplet acoustofluidic platform comprised a droplet generation device in polydimethylsiloxane (PDMS) bonded to an LN piezoelectric substrate on which interdigital transducers (IDTs) were patterned. To fabricate the PDMS device and IDTs, two photolithography masks were designed using LayoutEditor software. A flow-focusing junction was used in the PDMS device to generate microdroplets, which were collected in a 2800 μm by 2800 μm by 60 μm expansion chamber (Fig. 1A). IDTs were made of 10-nm Cr, 200-nm Au, and 1-nm Cr layers deposited on a 128° Y-cut LN substrate using the lift-off procedure. After the fabrication of IDTs, 300 nm of SiO_2_ was deposited on the LN substrate to protect the IDTs and enhance bonding to PDMS. Four IDTs were aligned at 45° with respect to the *x*-propagation direction of the LN substrate to ensure the same electromechanical coupling, and each IDT was arranged parallel with one side of the droplet expansion chamber. At each time, only two of the IDTs (opposite to each other) were activated. Soft lithography was used to fabricate the PDMS part. Hexamethyldisilazaneand AZ nLOF 2035 negative photoresist was spin-coated on a silicon wafer and then patterned to fabricate a master. The silicone elastomer base (SYLGARD 184, Dow Corning, MI, USA) was mixed with the curing agent (SYLGARD 184 silicone elastomer curing agent, Dow Corning, MI, USA) with a 10:1 ratio to fabricate the device using the master mold. Last, the PDMS part was aligned and bonded to the piezoelectric substrate using plasma.

### Experimental procedure

To generate droplets, the MFCS-EZ Fluigent system operating by MAESFLO software was used to control the inlet and outlet pressures. The prepared sperm sample was used as the main (middle) stream, and a biocompatible oil [Engineered oil (3M Novec) supplemented with surfactant (Pico-Surf 1, Sphere Fluidics, UK) to prevent merging] was used as the side stream at the flow-focusing junction to generate droplets. After generating sufficient droplets to fill the expansion chamber, atmospheric pressure was applied at the inlet and outlet ports to stop the flow and fix the droplets in the expansion chamber. Encapsulated sperm in droplets were imaged at 20 frames/s for 20 s in differential interference contrast imaging mode before, during, and after exposure to ultrasound for motility analysis. An inverted fluorescent microscope (Olympus IX83, Japan) equipped with an ORCA-Flash4.0 Digital complementary metal-oxide semiconductor camera (Hamamatsu Photonics, Japan) and a ×20 magnifications objective was used to capture the image sequences. SAWs were generated by applying a sinusoid electric signal using F20 powerSAW (Belektronig, Germany) to the IDTs at the frequency of 40 MHz and a power of 800 mW for 20 s. To monitor the device temperature, a forward-looking infrared (FLIR) thermal camera was used. The post-exposure videos were recorded for 5 s after turning off the acoustic field and when the acoustic streaming ceases.

### Motility analysis

To analyze sperm motility (fig. S1), the manual tracking plugin in ImageJ was used to track the position of sperm head and a custom MATLAB code (*48*) was used to calculate VCL, VAP, VSL, the ALH displacement, BCF, and LIN as defined by WHO (*79*). To measure VSL, sperm was tracked over a relatively short time period (*t* = 1 s) to minimize the influence of curved droplet boundaries (see fig. S7).

### Statistical analysis

Prism software (version 9) was used for statistical analysis to compare sperm motility parameters before and after exposure and also between different motility grades. Two-way analysis of variance (ANOVA) matched values with Bonferroni’s multiple-comparison test was used for within-grade group comparison to evaluate variations before and after exposure for each motility grade and for comparing immotile sperm stimulation results in Fig. 4. One-way ANOVA with Tukey’s multiple-comparison test was used for in-between group comparisons to evaluative statistical significance for changes between two motility groups. One phase decay least square fit nonlinear regression (at 95% confidence band) was used for the nonlinear trend analysis in Fig. 3. Linear regression (with 95% confidence) was used for linear trend analysis. Statistical significance for the MMP analysis (Fig. 5B) was determined using paired *t* test. *P* < 0.05 was considered significant (*P ≤ 0.05, **P ≤ 0.01, ***P ≤ 0.001, and ****P ≤ 0.0001).
