# Supplementary material for: Making immotile sperm motile using high-frequency ultrasound
Source: Sci Adv. 2024 Feb 14;10(7):eadk2864. doi: 10.1126/sciadv.adk2864 (PMC10866541; doi:10.1126/sciadv.adk2864)
Supplement: Supplementary file 1 — Figs. S1 to S7 Tables S1 to S7 Legends for movies S1 to S3 [file sciadv.adk2864_sm.pdf]

Science Advances
AAAS

Supplementary Materials for

Making immotile sperm motile using high-frequency ultrasound

Ali Vafaie *et al.*

Corresponding author: Reza Nosrati, reza.nosrati@monash.edu; Adrian Neild, adrian.neild@monash.edu

Sci. Adv. **10**, eadk2864 (2024)
DOI: 10.1126/sciadv.adk2864

The PDF file includes:

Figs. S1 to S7
Tables S1 to S7
Legends for movies S1 to S3

Other Supplementary Material for this manuscript includes the following:

Movies S1 to S3

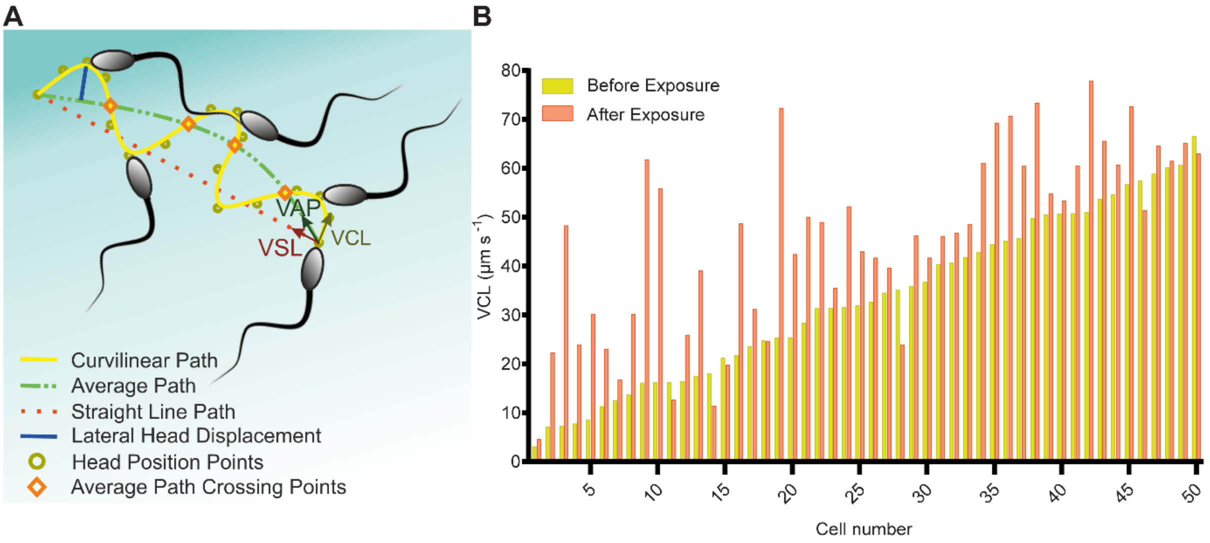

Fig. S1. Schematic of sperm motility parameters and sperm VCL pre- and post-exposure (**A**) Schematic of sperm swimming trajectory along its instantaneous swimming path (continuous yellow line) and the projected average path (green dash-dot line). Circles indicate sperm head position and orange lozenges show points at which instantaneous swimming path crosses the average path. Sperm motility parameters were defined as curvilinear velocity (VCL): sperm head point-to-point velocity in successive frames, average path velocity (VAP): projected sperm head point-to-point velocity in successive frames along its average path, straight line velocity (VSL): the velocity of sperm based on net displacement between the first and last tracking points, amplitude of lateral head displacement (ALH): the average deviation of the sperm head from the average path, beat cross frequency (BCF): the rate at which the sperm head crosses the average path, linearity (LIN): the ratio of VCL to VSL. (**B**) Comparison of the VCL values of 50 individual sperm cells (from three independent experiments using three biologically independent human samples) before and after exposure to ultrasound.

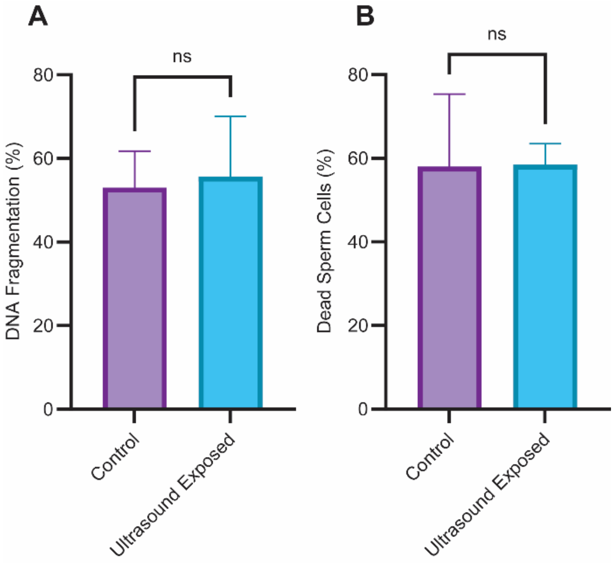

Fig. S2. **Biocompatibility of our ultrasound sperm motility boost platform.** (**A**) DNA fragmentation for exposed (n=621 sperm), and unexposed (control, n=437 sperm) samples, indicating no significant change in DNA integrity (P= 0.62). DNA fragmentation was evaluated using Acridine Orange staining. (**B**) Sperm viability for exposed (n=343 sperm) and unexposed (control, n=191 sperm) samples, indicating no significant change in viability (P= 0.93) post-exposure. Sperm from two biologically independent human samples in two independent experiments were used. Sperm viability was evaluated using LIVE/DEAD™ Sperm Viability Kit. Values are reported as mean ± s.d. (table S7). Statistical significance was determined using unpaired t-test.

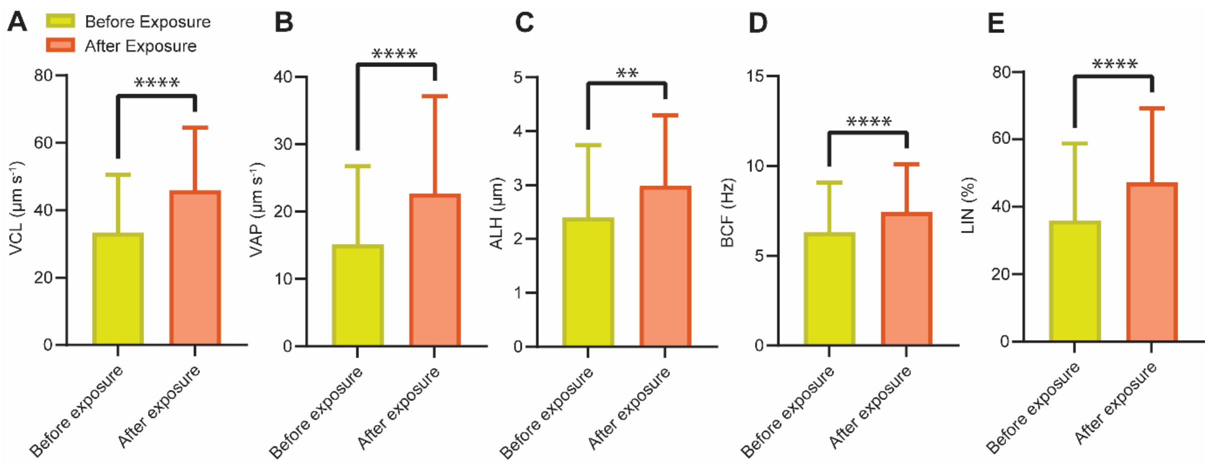

Fig. S3. **Boost to average motility after exposure**. Comparison of averaged (**A**) VCL, (**B**) VAP (**C**) ALH, (**D**) BCF and (**E**) LIN before and after exposure to ultrasound (n=50, from three independent experiments using three biologically independent human samples). Values are reported as mean ± s.d. Statistical significance was determined using paired t-test (* P ≤ 0.05, ** P ≤ 0.01, *** P ≤ 0.001, **** P ≤ 0.0001).

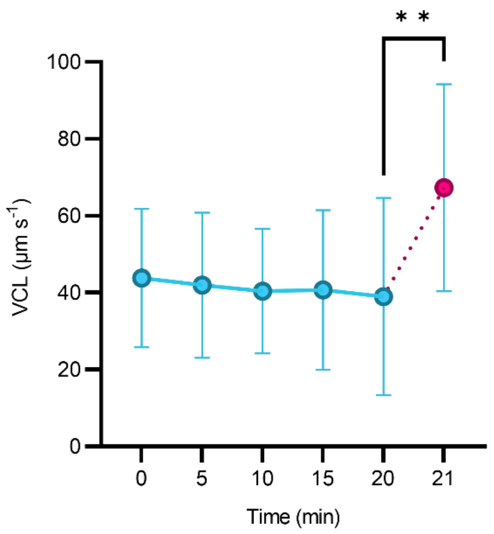

Fig. S4. Sperm VCL over 20 minutes pre-exposure (blue circles) compared to the post-exposure VCL (red circle). Values are reported as mean ± s.d. (n=20, from two independent experiments using two biologically independent human samples). Statistical significance was determined using one-way ANOVA with Tukey's multiple-comparison test (* P ≤ 0.05, ** P ≤ 0.01).

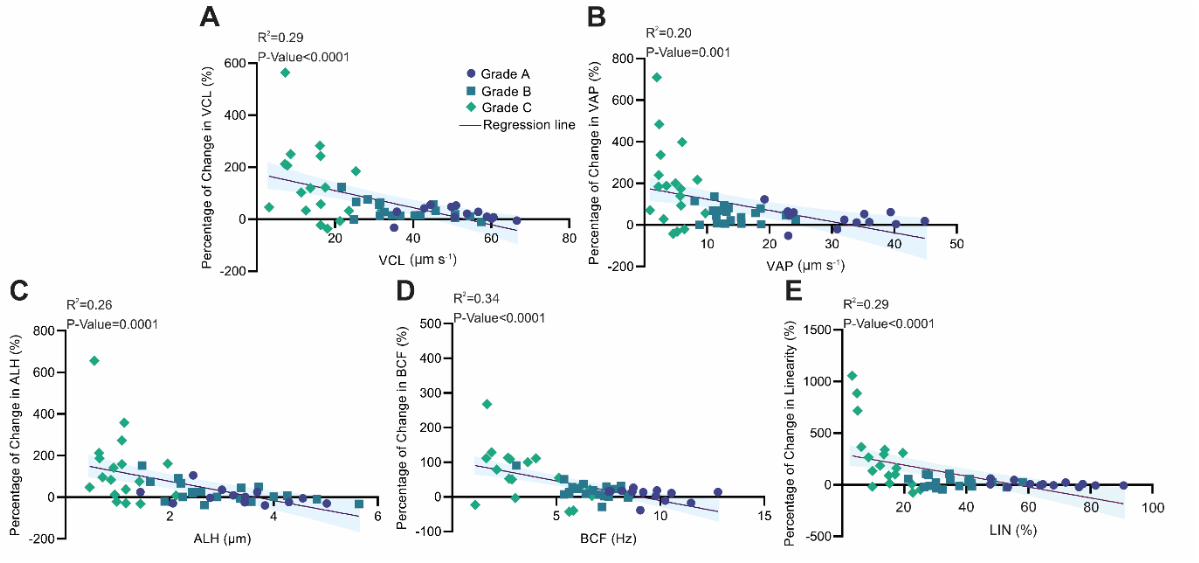

Fig. S5. **Single-cell representation of the percentage of change in motility parameters.** Percentage of change in motility parameters of each cell after exposure versus the initial values and corresponding linear regression for (**A**) VCL (R^2 =0.29, **** *P*<0.0001) (**B**) VAP (R^2 =0.20, *** *P*=0.001), (**C**) ALH (R^2 =0.26, **** *P*=0.0001), (**D**) BCF (R^2 =0.34, **** P<0.0001), and (**E**) LIN (R^2 =0.29, **** *P*<0.0001) (n=50, from three independent experiments using three biologically independent human samples). Highlighted area for each graph represents the 95% confidence band of the best-fit regression line.

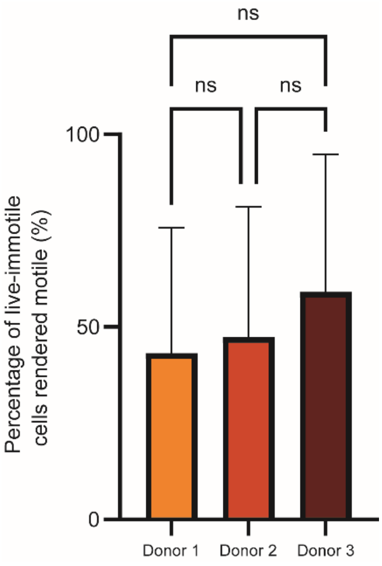

Fig. S6. **Percentage of live immotile sperm cells rendered motile after exposure for each donor.** n=33 for donor 1, n=30 for donor 2, and n=48 for donor 3. Values are reported as mean ± s.d. from six independent experiments. Statistical significance was determined using one-way ANOVA with Tukey's multiple-comparison test (ns denotes non-significant).

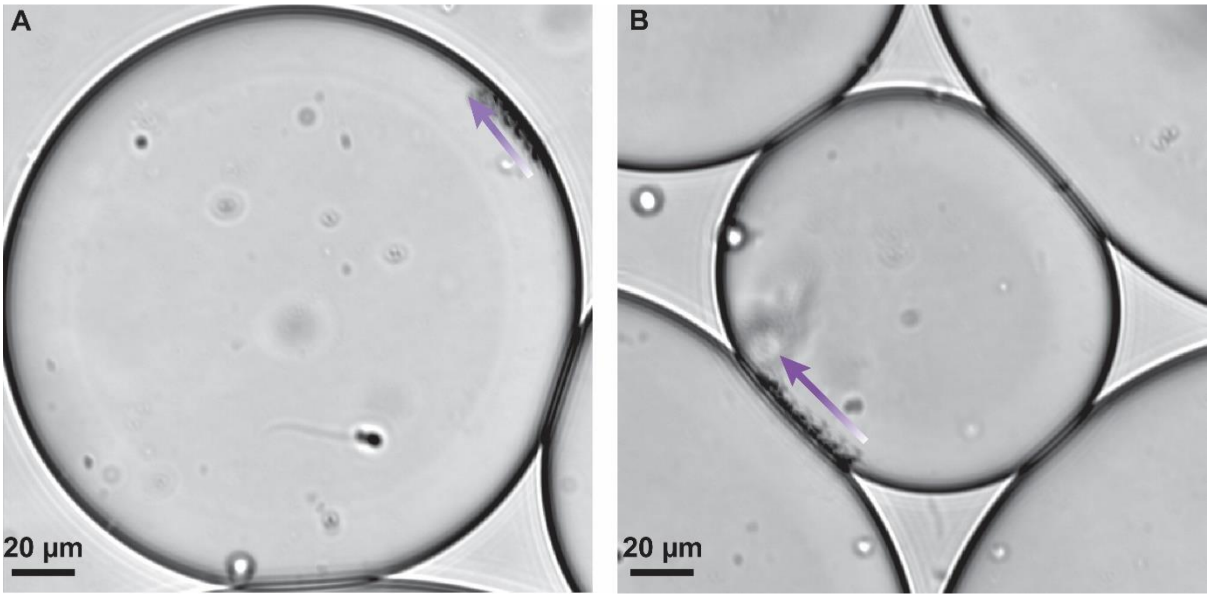

Fig. S7. Representative sperm trajectories used for the calculation of VSL. Overlaid images showing representative sperm trajectories for quantifying VSL in (**A**) a medium and (**B**) a small size droplet. Scale bar, 20 μm.

Table S1. Percentage of DNA fragmented and dead sperm in control and ultrasound-exposed groups. Values are reported as mean ± s.d.

	Control	Ultrasound -exposed
DNA fragmented sperm cells (%)	53.01±8.78	55.69±14.33
Dead sperm cells (%)	58.04±17.35	58.52±5.01

Table S2. Statistical significance of sperm motility analysis. *P* less than 0.05 was considered significant, **P* ≤ 0.05, ***P* ≤ 0.01, ****P* ≤ 0.001, *****P* ≤ 0.0001 and *ns* denotes not significant (n=50,38).

	**Analysis method**	**VCL ($\mu m\ s^{-1}$)**	**VAP ($\mu m\ s^{-1}$)**	**ALH (μm)**	**BCF (Hz)**	**LIN (%)**	**VSL ($\mu m\ s^{-1}$)**
Within-group comparison (Fig. 2A-E)	Two-way ANOVA matched values with Bonferroni's multiple-comparison test	Grade A: 1.92e-2 *	Grade A: 2.00e-4 ***	Grade A: >0.999 ns	Grade A: 6.53e-1 ns	Grade A: >0.999 ns	——
		Grade B: 2.90e-3 **	Grade B: 6.00e-4 ***	Grade B: 3.97e-1 ns	Grade B: 2.30e-2 *	Grade B: 1.34e-1 ns	
		Grade C: 1.00e-4 ****	Grade C: 6.00e-4 ***	Grade C: 3.00e-4 ***	Grade C: 5.00e-4 ***	Grade C: 1.00e-4 ****	
In-between group comparison (Fig. 2F-J)	Ordinary one-way ANOVA with Tukey's multiple-comparison test	Grades A&B: 9.32e-1 ns	Grades A&B: 8.98e-1 ns	Grades A&B: 8.75e-1 ns	Grades A&B: 7.67e-1 ns	Grades A&B: 9.67e-1 ns	——
		Grades A&C: 3.50e-3 **	Grades A&C: 2.20e-3 **	Grades A&C: 4.50e-3 **	Grades A&C: 7.90e-3 **	Grades A&C: 1.90e-3 **	
		Grades B&C: 3.80e-3 **	Grades B&C: 3.00e-3 **	Grades B&C: 7.50e-3 **	Grades B&C: 2.41e-2 *	Grades B&C: 1.40e-3 **	
Twitching group comparison (Fig. 4G)	Two-way ANOVA matched values with Bonferroni's multiple-comparison test	1.01e-6 ****	——	——	——	——	9.39e-1 ns
Progressive group comparison (Fig. 4H)	Two-way ANOVA matched values with Bonferroni's multiple-comparison test	3.12e-18 ****	——	——	——	——	5.79e-7 ****
Boost in average (fig. S3)	Paired t-test	1.15e-8 ****	2.65e-9 ****	2.1e-3 **	1.65e-5 ****	5.99e-5 ****	——

Table S3. Averaged boost in motility parameters at the population level (representing changes in averaged values) and single-cell level (representing average of changes for each individual sperm) referring to Fig. 2 and fig. S3. Values calculated at the population level represent the percentage of change in the averaged value of each motility parameter before and after exposure, calculated as $(\frac{\sum_{i=1}^{N} M_a(i)}{N} - \frac{\sum_{i=1}^{N} M_b(i)}{N}) \times 100/\frac{\sum_{i=1}^{N} M_b(i)}{N}$, in which $M_b(i)$ and $M_a(i)$ are the value of each motility parameter for i^{th} sperm before and after exposure, respectively, and N represents the total number of analyzed sperm cells. Values reported at the single cell level were calculated as $\sum_{i=1}^{N} \frac{M_a(i)-M_b(i)}{M_b(i)} \times 100/N$.

Motility parameter	Boost to average for all sperm cells (%)	Average boost to all sperm cells (%)	Boost to average for grade A sperm (%)	Average boost to Grade A sperm (%)	Boost to average for grade B sperm (%)	Average boost to Grade B sperm (%)	Boost to average for grade C sperm (%)	Average boost to Grade C sperm (%)
VCL (μm s^{-1})	37.7	66.3	19.9	20.6	27.3	32.4	108.7	135.0
VAP (μm s^{-1})	49.7	95.6	28.9	32.0	47.9	51.4	158.8	190.8
ALH (μm)	24.6	58.7	-0.9	4.2	14.2	23.3	112.1	135.3
BCF (Hz)	18.1	31.5	6.0	6.7	16.0	18.9	43.8	62.6
LIN (%)	30.5	93.0	4.5	7.0	22.1	24.23	153.3	265.9

Table S4. **Sperm motility parameters before and after exposure to ultrasound.** Values are reported as mean ± s.d.

	VCL (μm s^{-1})	VAP (μm s^{-1})	ALH (μm)	BCF (Hz)	LIN (%)	VSL (μm s^{-1})
Grade A, Within-group comparison (Fig. 2A-E)	Before exposure: 51.26±9.62	Before exposure: 31.13±8.07	Before exposure: 3.33±1.0	Before exposure: 9.50±1.51	Before exposure: 66.14±13.25	____
	After exposure: 61.48±13.94	After exposure: 40.14±13.13	After exposure: 3.30±1.1	After exposure: 10.07±2.09	After exposure: 69.44±14.59	____
Grade B, Within-group comparison (Fig. 2A-E)	Before exposure: 38.22±10.48	Before exposure: 14.36±4.33	Before exposure: 3.02±1.1	Before exposure: 6.61±1.33	Before exposure: 36.66±9.63	____
	After exposure: 48.65±10.36	After exposure: 21.31±6.87	After exposure: 3.45±1.1	After exposure: 7.67±1.45	After exposure: 44.75±17.11	____
Grade C, Within-group comparison (Fig. 2A-E)	Before exposure: 15.25±7.29	Before exposure: 4.53±2.38	Before exposure: 1.07±0.4	Before exposure: 3.68±1.86	Before exposure: 13.40±6.60	____
	After exposure: 31.82±18.36	After exposure: 11.66±7.82	After exposure: 2.29±1.4	After exposure: 5.29±2.20	After exposure: 33.94±18.87	____
Twitching group comparison (Fig. 4G)	Before exposure: 5.87±1.75	____	____	____	____	Before exposure: 0.41±0.20
	After exposure: 21.55±11.94	____	____	____	____	After exposure: 2.08±1.21
Progressive group comparison (Fig. 4H)	Before exposure: 8.39±4.05	____	____	____	____	Before exposure: 0.67±0.77
	After exposure: 44.92±19.50	____	____	____	____	After exposure: 16.43±7.53

Table S5. **Boost to sperm motility parameters after exposure to ultrasound.** Values are reported as mean ± s.d.

	Percentage of increase in VCL (%)	Percentage of increase in VAP (%)	Percentage of increase in ALH (%)	Percentage of increase in BCF (%)	Percentage of increase in LIN (%)
Grade A, Between-group comparison (Fig. 2F-J)	20.62±25.52	31.98±43.90	4.19±38.69	6.71±18.22	6.99±25.02
Grade B, Between-group comparison (Fig. 2F-J)	32.41±33.36	51.36±39.78	23.30±47.87	18.93±24.56	24.30±45.78
Grade C, Between-group comparison (Fig. 2F-J)	135.05±146.60	190.83±194.51	135.35±168.93	62.65±75.59	265.95±321.28

Table S6. Maximum percentage of increase in motility parameters versus pre-exposure VCL.

Pre-exposure VCL range	< 10 μm s^{-1}	10 to 20 μm s^{-1}	20 to 30 μm s^{-1}	30 to 40 μm s^{-1}	40 to 50 μm s^{-1}	50 to 60 μm s^{-1}	60 to 70 μm s^{-1}
Maximum boost to VCL (%)	564.6	283.1	185.1	65.1	56.5	52.7	7.5
Maximum boost to VAP (%)	710.2	398.8	218.1	138.1	123.9	65.5	19.5
Maximum boost to ALH (%)	656.4	358.4	161.7	67.0	105.7	36.5	-1.8
Maximum boost to BCF (%)	267.7	113.4	90.6	50.8	17.7	22.7	26.8
Maximum boost to LIN (%)	1057.5	309.6	340.5	108.2	93.4	63.0	47.9

Table S7. Ratiometric fluorescent intensity of sperm midpiece stained by JC-1. Values are reported as mean±s.d.

	Before exposure	After exposure
Ratiometric red to green fluorescent intensity (a.u.)	1.47±0.45	0.94±0.22

Supplementary movies

Supplementary Movie 1- **Representative sperm cells rendered motile after exposure to ultrasound.**

Supplementary Movie 2- **Example of a live immotile and a dead sperm responding to ultrasound.**

Supplementary Movie 3- **Representative immotile sperm rendered motile upon ultrasound exposure.**
